# Metastatic Choroiditis after the Extraction of a Molar Tooth

**Published:** 1883-11

**Authors:** 


### Metastatic Choroiditis After the Extraction of a Molar Tooth.

Dimmer, assistant to Prof. Arlt's clinic, reports a very interesting case; October 29th, 1883, a weak, anæmic looking boy was brought to the clinic with a history somewhat as follows: While at school, the patient accidently observed that he could see but very little with one eye, having the appearance of looking through a veil. The next day there was an apparent redness and swelling of the eye lids. Vision had disappeared entirely, and there was considerable exophthalmos. In the course of two weeks the swelling subsided somewhat; and a large quantity of pus was discharged from the orbit. Oct. 30th, both eye lids of the right eye were very much reddened and swollen, eye protruded to such an extent that the lids could not cover bulbus oculi. Movements of the eye ball are well preserved in every direction, the conjunctiva is very much injected; upward and inward there is a point as long as a pea covered with pus which proved to be an opening through the sclera and conjunctiva. Slight pressure causes pus to escape from this opening. Tension of the eye is somewhat lessened but it is not painful to the touch. Cornea has lost its lustre and is a little cloudy. After making thorough inquiry, the following was obtained: four weeks prior to his application he complained of toothache due to a carious tooth on the left side of the inferior maxilla, the tooth was at once extracted by a dentist. Two days later, a large painful swelling made its appearance on the left side, with pain in the throat and difficulty in deglutition. In two weeks pain and swelling left, but there were distinct chills two or three times during the day so that the patient was compeled to lie abed. The eye was bandaged, and in the course of a few days the cloudiness in the anterior chamber disappeared, so that the pupils could be distinctly seen. A distinct yellow reflex was plainly visible in the posterior chamber, with an opaque spot on the anterior surface of the capsule of the lens. The eye-ball diminished in size, and after three weeks, patient left the clinic, there was total synechia, posterior; pupil filled up with lymph, and granulations around the point of perforation through the sclera.

In this case, the pyæmia was evidently due to an injury to the lower maxilla received during the extraction of the carious tooth, but the mild course of the pyæmic process made it very difficult of recognition. TANGEMAN.

